# Case Report: Medicolegal evaluation in a case of fatal scald where a child fell into a cauldron in rural Nepal

**DOI:** 10.12688/f1000research.74607.2

**Published:** 2022-03-01

**Authors:** Alok Atreya, Lokaratna Gyawali, Ritesh G Menezes, Navneet Ateriya, Jamuna Shreshtha, Sristi Ghimire

**Affiliations:** 1 Department of Forensic Medicine, Lumbini Medical College, Palpa, 32500, Nepal; 2 District Hospital, Palpa, 32500, Nepal; 3 Forensic Medicine Division, Department of Pathology, College of Medicine, Imam Abdulrahman Bin Faisal University, Dammam, Saudi Arabia; 4 Department of Forensic Medicine & Toxicology, All India Institute of Medical Sciences, Gorakhpur, Uttar Pradesh, 273008, India; 5 Lumbini Provincial Hospital, Rupandehi, 32907, Nepal; 6 Lumbini Medical College, Palpa, 32500, Nepal

**Keywords:** Accident; fatal; Nepal; scald; thermal injury

## Abstract

Thermal injuries in young children involving the buttocks, perineum, and lower limbs raise suspicion of child abuse. Determining the manner of death and ruling out homicide in a fatal case of scalding remains a challenge for forensic practitioners. One such issue is reported in the present case report, where a medicolegal evaluation of a fatal scald in a two-year-old girl child is discussed. Young children sustaining serious injuries from scalds is a grave social concern. Such young lives need to be protected from scald injuries whether accidental or purposeful. Differences in injury patterns on the basis of their distribution and their characteristics are important to determine manner of death in such cases.

## Introduction

Thermal injuries from hot liquid are common cause of serious injuries to young children. Children are naturally curious about exploring their surrounding from a young age and are usually attracted towards steam from hot water. ^1^^,^^2^ Due to this, they are highly prone to develop superficial to deep scald injuries over body parts. The manner of such injuries may be accidental or homicidal.

Thermal injuries in children are preventable; however, ignorance and neglect may lead to a mishap and, in extreme cases, a fatality. Accidental scalds are a common form of thermal injuries which usually occur in home-settings. ^1^^–^^5^ Children under five years of age sustain ten times more thermal injuries than children above five years. ^4^ Scald injuries affect younger children on a large scale. Sometimes, it is very difficult to differentiate whether the injuries inflicted on the body are because of an accident or homicidal act. Thus, it is very important to rule out all possibilities before arriving at a conclusion in these cases. The present case describes a scenario from rural Nepal where a two-year-old girl child allegedly fell into a cauldron where animal feed was being stewed and sustained fatal scald injuries. The event was unwitnessed, and a history was given by the family members on the basis of circumstances. The present article further discusses the pattern and the nature of such injuries sustained by the victim.

## Case report

### Case history

It was the Nepalese New Year (mid-April), and the school was closed for vacation. The two-year-old daughter, accompanied by her mother had come to her maternal grandparents’ house, in a mountainous village of rural Nepal. On the evening in question, the individuals present in the house were the maternal grandmother, maternal grandfather, mother, and the victim. It was early in the evening and the family had left cattle feed to stew on a cauldron. As it would take around an hour to cook, it was usual for the family to leave it unattended and engage in other chores. On the fateful evening at around 1900 hours, the victim accidently fell into the cauldron while playing. Her grandfather carried her from the scene of incident to the nearest health post about a kilometre away. Looking at the grave nature of the injury the health assistant at the health post referred her to a larger centre with a burn care unit as the rural health post had a limited facility. At this point, the victim was given paracetamol suspension for pain relief. No other treatment or wound care was provided in the health post. An ambulance was called, and she was rushed to a hospital in Palpa. It was three-hour drive and she reached the hospital at around 2300 hours. As per the hospital records, the child was restless and crying in agitation when presented to Emergency Department (ED). She was afebrile, her body temperature was 98.5 Fahrenheit (F), pulse 126 beats per minute, respiration 22 per minute, and oxygen saturation was 95% at room air. A quick general survey done estimated 50-55% deep dermal burn involving front and back of chest, both upper limbs and back of the both thighs involving buttocks. There was superficial burn noted around the head neck region and front of abdomen and front of both the thighs. Under Ketamine anesthesia, the wound was thoroughly cleaned with normal saline, 1% silver sulfadiazine cream was applied evenly over the burn area and dressing was done. The child was given tetanus immunization. Intravenous line (IV) was secured and the Ringer’s lactate solution was started. Prophylactic broad-spectrum antibiotics (IV) and pain medication was started. Catheterization was done with Foley’s. Dressing was planned on every alternate day. In the evening of third day of admission, the child was restless, and the temperature was 100.8 F. The fever spiked to 102.4 F on the following morning. Blood and wound swabs were sent for culture and one more antibiotic was added for broad coverage. On day five, the child was afebrile. On the evening of day six, the child was restless, with oxygen saturation dropping to 85-87%.

Despite treatment upon arrival, she succumbed to the complications on the seventh day of admission. A police inquest was done, and the body was then subjected to a post-mortem examination. As per the Nepalese law, it is mandatory to conduct medicolegal examination in all cases of unnatural deaths. The post-mortem was conducted at the district hospital, Palpa.

### Autopsy

**Figure 1.**
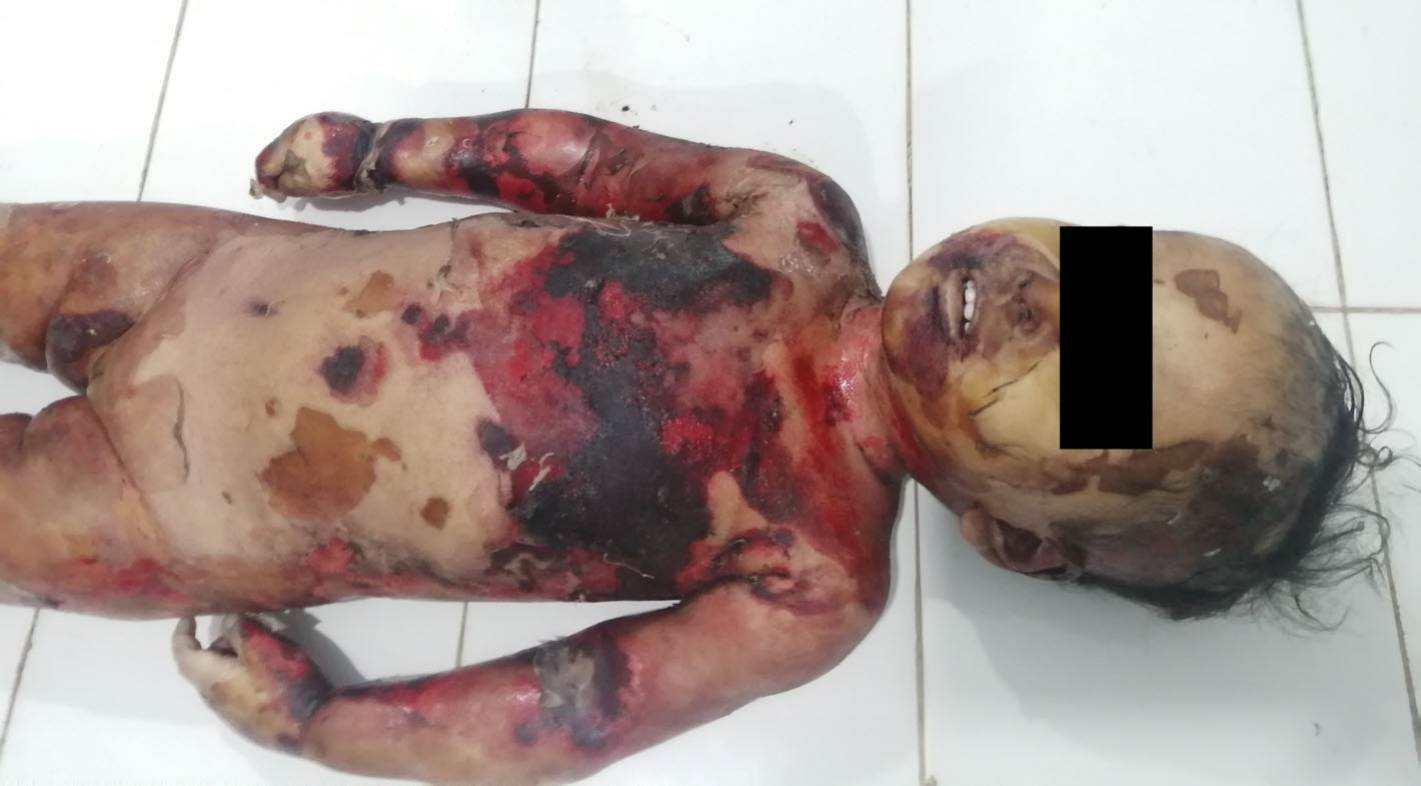
The front of the body showing the scalded area.

**Figure 2.**
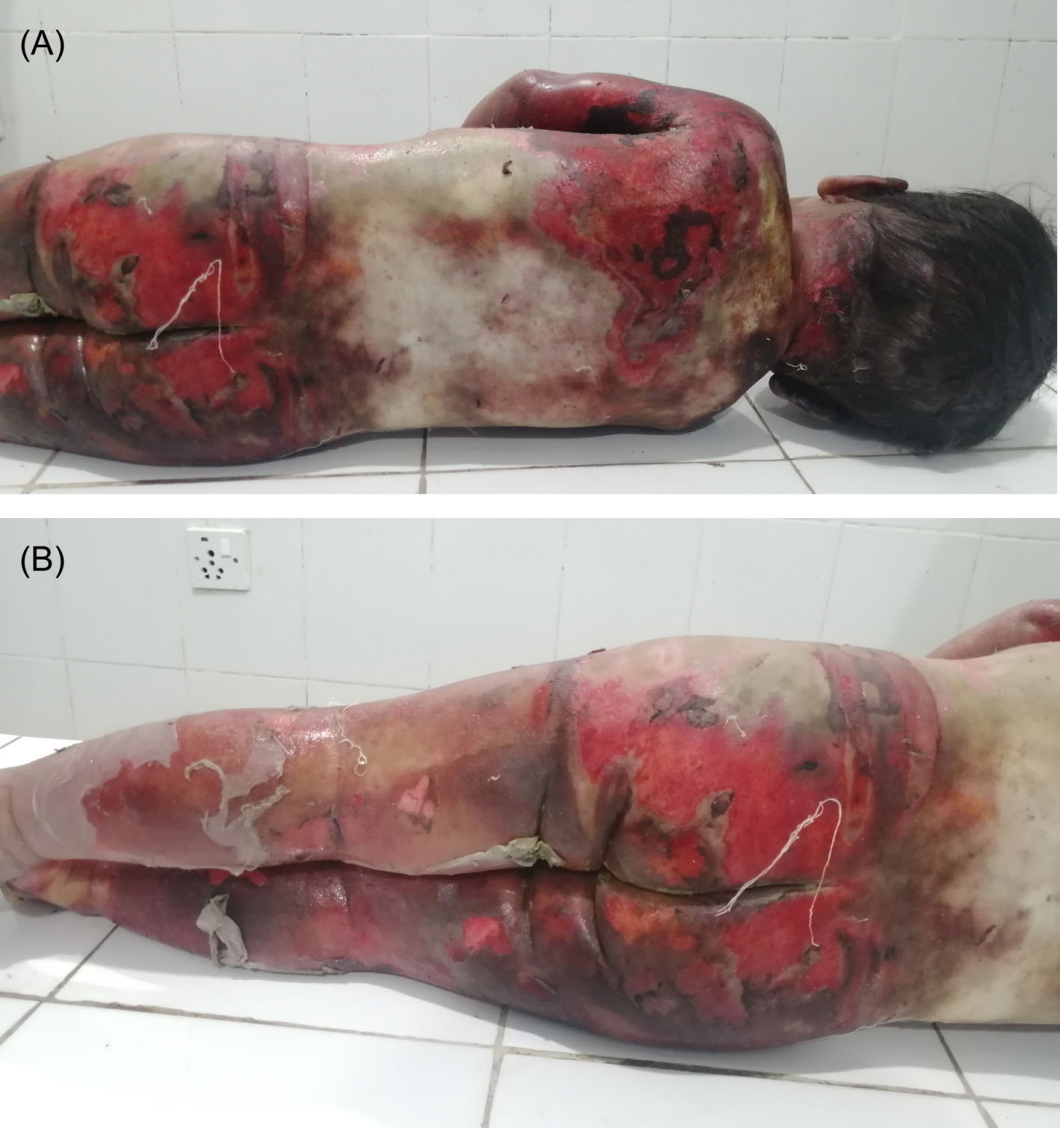
A and B. The back of the body and lower limbs showing the area involved in scald.

The deceased was a female of weight 13.5 kg and length 80 cm. External examination revealed superficial to deep scald injuries present over the body involving about 90% of the total body surface area ( Figure 1). Peeling of skin was observed over the body in several places, most notably on both lower limbs ( Figure 2A and B). Yellowish discoloration of skin was observed over the mid-back and shoulders with the presence of foul-smelling wounds and unhealthy granulation tissue as a result of infected wounds. Reddish, healthy granulation tissue was also observed over the body at places. Hairs present over the scalp and other body parts did not show any sign of singeing. No other injury was noted over the body. Upon internal examination, organs were grossly congested. Otherwise, no remarkable finding was observed. The cause of death was opined as complications of scald injuries sustained involving about 90% of the total body surface area.

### Scene of the incident

As per the history provided during the police inquest by the family members, the victim accidently fell into the cauldron where the cattle feed was being stewed for the domesticated buffalo. The cattle feed is generally cooked in the cauldron without a cover. Medicolegal autopsies in Nepal follow police inquests. Expedite autopsy and quick report mentioning the cause of death is primarily what the investigating officer is interested in and therefore a visit to the scene of death by the doctor performing the autopsy is usually not undertaken. Death scene photographs are also not obtained in most of the cases. In this case, the investigating officer did not photograph the scene. History was taken from the relatives by the investigating officer as a part of the full investigation via interviews with all members present in the house. Certain details regarding the scene were not available, including size, weight, and placement of the cauldron. However, it was unlikely that the victim could climb and enter into the cauldron, because the burning firewood and the heated cauldron would not allow the victim to climb up it. It was further unknown if the cauldron was still over the fireplace or it was moved down from the clay stove. Another possibility might be that the victim might have accidentally knocked the cauldron while playing and spilled the broth all over her body. The spillage which was on the ground might have caused deeper scald over the back of their legs and the buttocks as noted in the autopsy. A remote possibility of deliberate immersion of the child in hot liquid cannot be ruled out. When the mother was questioned on how the child was injured as part of the police inquest, she replied saying the child fell into the cauldron. However, when she was asked if the child fell into the cauldron or cauldron fell on the child, she said she was not present at the scene of incident and arrived only after she heard the cry of her child.

## Discussion

Scald injuries are an important public health issue worldwide. Most of the cases of thermal injuries are reported from the low and middle-income countries around the globe. ^6^ Although most scald injuries are preventable, when sustained they cause significant morbidity and mortality to victims. ^7^ Younger children are more curious in exploring their surroundings making them prone to suffer variety of injuries. Hence, ensuring that they are not left alone while they are doing their routine activity may help to prevent any such kind of catastrophic event. Sustaining scald injuries even on a smaller area of body may also cause sufficient damage to the skin of young children.

Almost 70% of all thermal injuries in infants, toddlers, and pre-school children are due to scalds and mostly occur at home. ^8^ Shah *et al.,* reported that the risk for scald injury was greatest in children of 13 to 24 months of age. ^7^ This is most likely because of their dependency on the mothers and increasing mobility, thus involving more risk of getting injured by spillage of hot liquid or other materials present in the household. ^8^ A study done by Prabakaran revealed a slightly higher proportion of females (53%) compared to males (47%) in the study population. ^9^ A similar trend where the female to male ratio was more and was also observed in the studies conducted by Shah *et al.,* (1.22:1), Rimmer *et al.,* (1.08:1), Yeoh *et al.,* and Agbenorku *et al.* ^7^^,^^10^^–^^12^ The World Health Organisation states that adult females have slightly higher death rates from thermal injuries than males because of open fire cooking, unsafe cookstoves, and loose clothing. ^6^ This is opposite to the usual injury pattern sustained by the victims, where rates of injuries are higher in males than females. ^6^ Children too are vulnerable to thermal injuries as adult women as children accompany their mothers most of the times. While improper adult supervision is considered as a major risk factor for childhood thermal injury, child maltreatment too should not be overlooked. ^6^

Medicolegal examination of victims of thermal injury is a challenge in forensic practice. ^13^ In cases of scalds in children, there is suspicion if the injuries were nonaccidental in nature. Thermal injuries in children involving buttocks and lower limbs point more towards abuse. ^14^ Russo *et al.,* in 1986 suggested some criteria to differentiate between nonaccidental and accidental injuries. ^15^ The following features are suggestive of child abuse: if the history is inconsistent with the physical findings, when there is a delay in seeking of medical attention following injury, when there are multiple blunt force injuries with different stages of healing, and when there is localized burn involving the buttocks, genitalia, or perineum. ^14^^,^^15^ If a child is brought to the hospital by people other than the caretakers, it can also raise suspicions of abuse. ^7^ The circumstances of injury should also corroborate the autopsy findings. ^12^ In the present case, the manner of injury could not be established as outlined in the history.

Thermal injuries are fatal specially in children, with most deaths occurring during the first week after injury. ^16^ In the present case, the victims died in the hospital a week after sustaining scald injury. The cause of death in fatal cases is usually due to complications of thermal injuries sustained, with the most common complications being septicaemia, hypovolemic shock, neurogenic shock, and multiple organ failure. ^17^ In such cases, timely management is important especially the response from the first responder in the form of providing first aid at the site of incident itself. Upgrading the rural primary health care centres and appointment of medical doctors would save time and lives in serious injuries in remote rural locations of a mountainous country like Nepal. In present case, first aid was not provided at the scene or initial health centre, and there was a delay in treatment as the victim was transferred. The government should frame a policy in this regard. Furthermore, campaigns for awareness can be conducted through radio, television, and in the form of pamphlets, brochures, and street plays. Such campaigns would make the general public aware in preventing unintentional injuries in younger children.

There were some limitations noted in the examination of the present case. Circumstantial evidence, death scene visits, and death scene photographs are important while considering the investigation into the manner of death. In the present case, a detailed death scene investigation and photographic evidence were not available. The police inquest mentioned that the child fell into the cauldron while playing with no additional analyses. As seven days had already passed and the victim died while under treatment, police did not visit the actual death scene and the inquest was done in the hospital itself. The police inquest detailed the location of the mortuary cold chamber where they viewed the dead body. The bereaved family were also reluctant to provide the details of the incident due to fears of the medicolegal nature of the case and potential legal hassle.

## Conclusion

Unintentional thermal injuries are a great burden to the community, especially in the case of children. This should be considered as an essential public health issue. Most thermal injuries are preventable; educating the caretakers might help reduce further thermal injury-related morbidity and mortality.

## Data availability

All data underlying the results are available as part of the article and no additional source data are required.

## Consent

Written informed consent for publication of the clinical details and/or clinical images was obtained from the deceased’s mother.
