# Supplementary material for: Case Report: Medicolegal evaluation in a pediatric case of fatal scald injury from rural Nepal
Source: F1000Res. 2022 Mar 16;11:35. Originally published 2022 Jan 12. [Version 3] doi: 10.12688/f1000research.74607.3 (PMC8917323; doi:10.12688/f1000research.74607.3)
Supplement: Peer review history

# Peer review: Reviewer response for version 3

**DOI:** 10.5256/f1000research.122447.r127755

**Published:** 2022-03-17

**Authors:** Wasey Ali Yadullahi Mir

**Affiliations:** 1 Department of Internal Medicine, Mount Sinai Hospital, Chicago, IL, USA

Good. Approved

Are enough details provided of any physical examination and diagnostic tests, treatment given and outcomes?

Partly

Is the case presented with sufficient detail to be useful for other practitioners?

Partly

Is sufficient discussion included of the importance of the findings and their relevance to future understanding of disease processes, diagnosis or treatment?

Yes

Is the background of the case’s history and progression described in sufficient detail?

Yes

Reviewer Expertise:

Internal Medicine, Critical Care, Pulmonology and Cardiology

I confirm that I have read this submission and believe that I have an appropriate level of expertise to confirm that it is of an acceptable scientific standard.

# Peer review: Reviewer response for version 2

**DOI:** 10.5256/f1000research.121928.r125831

**Published:** 2022-03-11

**Authors:** Biliana Mileva

**Affiliations:** 1 Department of Forensic Medicine and Deontology, Medical University of Sofia, Sofia, Bulgaria

Thank you for making the effort to revise the article and for adding more detailed information concerning the case. I believe that the article should be published in its current form.

Are enough details provided of any physical examination and diagnostic tests, treatment given and outcomes?

Partly

Is the case presented with sufficient detail to be useful for other practitioners?

Partly

Is sufficient discussion included of the importance of the findings and their relevance to future understanding of disease processes, diagnosis or treatment?

Yes

Is the background of the case’s history and progression described in sufficient detail?

Partly

Reviewer Expertise:

Forensic medicine, Autopsy, Forensic pathology,

I confirm that I have read this submission and believe that I have an appropriate level of expertise to confirm that it is of an acceptable scientific standard.

# Peer review: Reviewer response for version 2

**DOI:** 10.5256/f1000research.121928.r125833

**Published:** 2022-03-02

**Authors:** Wasey Ali Yadullahi Mir

**Affiliations:** 1 Department of Internal Medicine, Mount Sinai Hospital, Chicago, IL, USA

Thank you for sending me the revised editions. I believe the authors have done a good job in revising and providing the requested information. Unfortunately, certain things cannot be written in more detail due to a lack of police investigation. I believe it should be accepted and move further.

Are enough details provided of any physical examination and diagnostic tests, treatment given and outcomes?

Partly

Is the case presented with sufficient detail to be useful for other practitioners?

Partly

Is sufficient discussion included of the importance of the findings and their relevance to future understanding of disease processes, diagnosis or treatment?

Yes

Is the background of the case’s history and progression described in sufficient detail?

Yes

Reviewer Expertise:

NA

I confirm that I have read this submission and believe that I have an appropriate level of expertise to confirm that it is of an acceptable scientific standard.

# Peer review: Reviewer response for version 1

**DOI:** 10.5256/f1000research.78382.r120278

**Published:** 2022-02-07

**Authors:** Biliana Mileva

**Affiliations:** 1 Department of Forensic Medicine and Deontology, Medical University of Sofia, Sofia, Bulgaria

This article presents a rare case in the medico-legal practice. It refers to a two-year-old girl who sustained a severe thermal injury - scalds in unclear circumstances and dies a week later. Scalds are commonly seen injuries but rarely fatal. Since scalds are a well-known method of child abuse - a careful examination and evaluation of the sustained injuries over the victim's body are needed. In the case presented, there is a lack of information concerning the scene of the incident. The above makes the evaluation and interpretation of the sustained injuries of great importance. The authors have summarized the subject adequately, but more details would make it perfect. Maybe consider adding:

1. More information concerning the condition of the girl on admission to the hospital, the treatment during the hospital stay, and the occurred complications.
2. A more detailed description of the distribution of the injuries over the body - if there are any intact areas, the degree of burns in different anatomical areas, etc., and their interpretation.

Are enough details provided of any physical examination and diagnostic tests, treatment given and outcomes?

Partly

Is the case presented with sufficient detail to be useful for other practitioners?

Partly

Is sufficient discussion included of the importance of the findings and their relevance to future understanding of disease processes, diagnosis or treatment?

Yes

Is the background of the case’s history and progression described in sufficient detail?

Partly

Reviewer Expertise:

Forensic medicine, Autopsy, Forensic pathology,

I confirm that I have read this submission and believe that I have an appropriate level of expertise to confirm that it is of an acceptable scientific standard, however I have significant reservations, as outlined above.

# Response

**Published:** 2022-02-28

**Authors:** Navneet Ateriya

We thank the learned reviewer for their valuable time going through our manuscript. We have addressed all the suggestions made by the reviewers.

*Comment: This article presents a rare case in the medico-legal practice. It refers to a two-year-old girl who sustained a severe thermal injury - scalds in unclear circumstances and dies a week later. Scalds are commonly seen injuries but rarely fatal. Since scalds are a well-known method of child abuse - a careful examination and evaluation of the sustained injuries over the victim's body are needed.*

 Response: Thank you.

*Comment: In the case presented, there is a lack of information concerning the scene of the incident. The above makes the evaluation and interpretation of the sustained injuries of great importance.*

 Response: We do agree with the reviewer that the case presented lacks much detail on the scene of incident. The investigating officers of the present case thought this to be a case of an accident and did not visit the crime scene for further investigation. Due to the lack of details of the scene of incidence, there was difficulty in interpreting the manner of death in the present case, which was the focus of the present study and this has been discussed in the discussion section.

*Comment: The authors have summarized the subject adequately, but more details would make it perfect. Maybe consider adding:*

- *More information concerning the condition of the girl on admission to the hospital, the treatment during the hospital stay, and the occurred complications.*
- *A more detailed description of the distribution of the injuries over the body - if there are any intact areas, the degree of burns in different anatomical areas, etc., and their interpretation.*

Response: This information is added.

# Peer review: Reviewer response for version 1

**DOI:** 10.5256/f1000research.78382.r119779

**Published:** 2022-01-21

**Authors:** Wasey Ali Yadullahi Mir

**Affiliations:** 1 Department of Internal Medicine, Mount Sinai Hospital, Chicago, IL, USA

The authors have presented a case of a female child who presented with extensive burns on admission causing death within 7 days. The autopsy revealed death due to complications of scald injuries. Also puts forth the details of police inquest and lack of emergent care provided at a rural location and delay in treatment in the burn center. Unfortunately, the manner of death was not determined due to reasons provided in the case. The authors also provide the major issue of lack of awareness leading to such preventable causes of injury.

 The authors did provide the images, however, the details on the child's presentation on arrival, complications of burns, and seven days treatment in hospital are not discussed in detail.  Maybe considering adding a few more details on how to differentiate child abuse in immersion burns from accidental burns in forensic practice will be helpful for readers in the discussion part of the paper.

Are enough details provided of any physical examination and diagnostic tests, treatment given and outcomes?

Partly

Is the case presented with sufficient detail to be useful for other practitioners?

Partly

Is sufficient discussion included of the importance of the findings and their relevance to future understanding of disease processes, diagnosis or treatment?

Yes

Is the background of the case’s history and progression described in sufficient detail?

Yes

Reviewer Expertise:

Pulmonary disease, Critical Care Medicine, Cardiology and Internal Medicine

I confirm that I have read this submission and believe that I have an appropriate level of expertise to confirm that it is of an acceptable scientific standard, however I have significant reservations, as outlined above.

# Response

**Published:** 2022-02-28

**Authors:** Navneet Ateriya

We thank the learned reviewer for their valuable time going through our manuscript. We have addressed all the suggestions made by the reviewers.

*Comment: The authors have presented a case of a female child who presented with extensive burns on admission causing death within 7 days. The autopsy revealed death due to complications of scald injuries. Also puts forth the details of police inquest and lack of emergent care provided at a rural location and delay in treatment in the burn center.*

*Unfortunately, the manner of death was not determined due to reasons provided in the case. The authors also provide the major issue of lack of awareness leading to such preventable causes of injury.*

 Response: Thank you.

*The authors did provide the images, however, the details on the child's presentation on arrival, complications of burns, and seven days treatment in hospital are not discussed in detail.*

*Maybe considering adding a few more details on how to differentiate child abuse in immersion burns from accidental burns in forensic practice will be helpful for readers in the discussion part of the paper.*

 Response: We have added the details on the child's presentation on arrival at the hospital as per the available hospital records. The points to differentiate accidents from intentional scalds are discussed in the discussion section.
